# Physical function in patients newly diagnosed with multiple myeloma; a Danish cohort study

**DOI:** 10.1186/s12885-020-6637-6

**Published:** 2020-03-03

**Authors:** Rikke Faebo Larsen, Mary Jarden, Lisbeth Rosenbek Minet, Ulf Christian Frølund, Sören Möller, Niels Abildgaard

**Affiliations:** 1 Department of Physiotherapy and Occupational Therapy, Zealand University Hospital, Roskilde, Denmark; 2 Department of Clinical Research, University of Southern Denmark, Odense, Denmark; 3 OPEN, Open Patient data Explorative Network, Odense University Hospital, Odense, Denmark; 4 Department of Haematology, Copenhagen University Hospital, Rigshospitalet, Copenhagen, Denmark; 5 Department of Public Health, Faculty of Health and Medical Sciences, University of Copenhagen, Copenhagen, Denmark; 6 Department of Rehabilitation, Odense University Hospital, Odense, Denmark; 7 Health Science Research Centre, UCL University College, Odense, Denmark; 8 Department of Haematology, Zealand University Hospital, Roskilde, Denmark; 9 Department of Haematology, Odense University Hospital, Odense, Denmark; 10 The Academy of Geriatric Cancer Research (AgeCare), Odense University Hospital, Odense, Denmark

**Keywords:** Multiple myeloma, Bone disease, Physical function, Reference values, Cross sectional

## Abstract

**Background:**

Multiple myeloma is a cancer in the bone marrow causing bone destruction. Patients experience various symptoms related to the disease and/or treatment, such as pain and fatigue, leading to poorer quality of life. The symptom burden might affect physical function and physical activity levels, posing a risk of physical deterioration. The aim was to investigate whether physical function in newly diagnosed patients with multiple myeloma differs from the reference values of the normal population and other cancer patients.

**Methods:**

The study is a cross sectional descriptive analysis of a prospective cohort of 100 patients newly diagnosed with multiple myeloma. Four physical function tests were carried out; Six-Minute-Walk-Test, Sit-to-Stand-Test, grip strength and knee extension strength. Age and gender specific results of physical function from the multiple myeloma population were compared to normative data and to data from other cancer populations.

**Results:**

Of the 100 patients included, 73% had bone disease and 55% received pain relieving medicine. Mean age was 67.7 years (SD 10.3). Patients with multiple myeloma had significantly poorer physical function compared to normative data, both regarding aerobic capacity and muscle strength, although not grip strength. No differences in physical function were found between patients with multiple myeloma and other cancer populations.

**Conclusions:**

Physical function in newly diagnosed Danish patients with multiple myeloma is lower than in the normal population. Exercise intervention studies are warranted to explore the value of physical exercise on physical function.

**Trial registration:**

ClinicalTrials.gov, ID NCT02439112, registered 8 May 2015.

## Background

Multiple myeloma (MM) is a plasma cell cancer in the bone marrow that primarily affects older adults. In Europe the incidence of MM is 5.72 per 100,000, and the median age at diagnosis is 68 years [1, 2]. A hallmark of MM is the associated bone disease, which includes bone destructions, vertebral collapses and other pathological bone fractures, and hypercalcemia. Bone involvement is seen in about 79% of newly diagnosed patients with MM [3]. In addition, anemia is common, presenting in approximately 73% of patients with MM [3]. Patients newly diagnosed with MM report low quality of life and reduced physical function, and pain and fatigue are dominant symptoms [4–7]. Moreover, patients with MM experience a greater symptom burden and more severe symptoms than patients with other malignant haematological diseases, negatively affecting their quality of life, especially, role, physical, and social function [8]. At time of diagnosis, global quality of life is affected and all five functional scales (physical, role, social, emotional, and cognitive functioning) on EORTC are negatively affected. Pain and fatigue are the most prevalent and distressing symptoms [9, 10].

Physical fitness, including endurance, strength, flexibility, and balance, is associated with physical function, physical functional limitation and physical independence [11, 12]. Physical indicators, such as low level of physical activity, lower extremity function, and low grip strength can predict disabilities related to activities of daily living, e.g. walking, transferring, bathing or dressing [13]. Mobility limitations 30 days after discharge among older medical patients can be predicted by measurements of handgrip strength, gait speed, modified chair stand test and the Cumulated Ambulation Score, where chair stand test (Sit-to-Stand-Test) and gait speed are the strongest predictors [14]. Thus, both aerobic capacity and strength are important for physical function in daily life, not least in the older population, since physical fitness is associated with age [11, 12], and improved physical function may positively affect quality of life.

Though not being the only determining factor, physical function contributes significantly to the performance status of a patient, exemplified when the Eastern Cooperative Oncology Group (ECOG) performance status of a patient is assessed. In patients with MM, affected ECOG performance status, particularly performance status 3–4, is a major predictor of an adverse prognosis [15, 16].

In spite of the bone destructive nature of MM and well described low patient-reported physical function levels, we have not been able to identify studies that report the objective physical function among newly diagnosed patients with MM. By testing physical function, patients at risk could be identified, and interventions to prevent physical deterioration or improve physical function could be initiated. Maintaining or improving physical function is fundamental for the patients to carry out usual activities and in maintaining their quality of life [17, 18]. The effect of exercise in cancer patients is well documented [19], as well as in the elderly [20]. In patients with MM physical training has been shown to be safe and feasible [21, 22]. Furthermore, knowledge about whether and how patients with MM differ from other cancer populations would be helpful for clinical practice in the planning of exercise interventions.

We hypothesised, that patients with MM have poorer physical function than the normal population and patients with other cancer diagnoses. The aim of this study was to describe age and gender specific physical function among patients newly diagnosed with multiple myeloma and to compare physical function to the normal population and other cancer populations.

## Methods

This is a cross sectional, descriptive analysis of a cohort of 100 patients with newly diagnosed MM. The patients were prospectively and consecutively included at two departments of haematology at two University Hospitals in Denmark from 22 June, 2015 to 18 January, 2019 as part of a randomised, controlled trial (ClinicalTrials.gov., ID NCT02439112) investigating the effect of a 10 week exercise intervention. Patients were screened for eligibility at time of diagnosis by the haematologist, based on inclusion and exclusion criteria. Introductory information about the study was given, and afterwards the principal investigator contacted the patient to give further information and for final inclusion. Included were patients ≥18 years of age newly diagnosed with treatment demanding MM (High Dose Therapy with Stem Cell Transplantation (HDT-SCT) or less intensive treatment), and who were able to speak and understand Danish. Exclusion criteria were spinal cord compression, unstable vertebral fracture (Spinal Instability Neoplastic Score > 12) [23], untreated cardiac failure or untreated cardiac arrhythmia, severe chronic cardiac failure (NYHA 3–4), other severe comorbidity that according to treating physician would not permit physical exercise, and psychological or psychiatric disorders. Written informed consent are obtained from all individual participants included in the study.

### Data collection

Prior to start of the treatment in an outpatient setting, all eligible patients were tested with the following physical function measurements: Six-Minute-Walk-Test (6MWT) [24] as a functional measure of aerobic capacity, Sit-to-Stand-Test (SST) [25] as a functional measure of lower body strength, grip strength [26, 27] as a measure of upper body strength and a direct measure of isometric knee extension strength [27, 28]. Prior to testing, the haematologist performed a systematic assessment of the impact of the radiologically assessed bone disease to determine restrictions regarding the physical tests (and exercise as well, to be used in the randomised controlled trial). In relation to testing, our focus was on the femoral bone. The assessment captured size of osteolytic lesions, fractures, and if applicable, estimated the time of fractures, and the haematologist assessed the degree of pain. Based on Mirel’s scoring system [29], this combined information of location, fractures/size of lesions and pain were used to assess whether the fractures and/or bone destructions should restrict certain tests. That was the case if an osteolytic lesion in the femoral bone involved between one third and up to two thirds of the diameter and caused pain, *or* if an osteolytic lesion involved more than two thirds of the diameter or involved the cortical bone (cortical thinning), even without associated pain. In these cases we only tested the unaffected side and omitted SST.

The physical function data (6MWT, SST, grip strength and knee extension strength) used in the current analysis are data from the baseline measures in the randomised controlled trial (ClinicalTrials.gov, ID NCT02439112), conducted by a project team of trained physiotherapists. Patient demographic and medical characteristics were collected from the patients’ medical records. The testing of 6MWT, SST and grip strength followed guidelines [25, 27, 30–33], while the knee extension strength was measured by a standardised protocol developed for the randomised controlled trial. It was measured by a dynamometer (Lafayette Manual Muscle Tester), which was perpendicularly fixated to a bench by a strap. The participant was sitting on the bench with hip and knee flexion of 90° and arms resting on the side. Then the strap with the dynamometer was placed around the participant’s lower leg. The lower border of the dynamometer was placed five centimeters from the top of the lateral malleolus. The patient had three measures of right and left side, respectively regarding grip strength (until maximum value) and knee extension strength (each try was 5 s). The highest value was used for the analysis.

We included normative data of physical function outcomes from different healthy populations [34–36] and published data from other cancer disease populations; malignant lymphoma before starting chemotherapy and without bone metastasis or elevated risk of fracture [37], prostate cancer after surgery or radiotherapy [38, 39] and breast cancer post-treatment [40, 41]. These cancers were chosen to compare MM data to other haematological cancers, both malignant lymphoma without bone destructions, and solid cancers where bone destructions are common. Comparison with other haematological cancers is relevant, because typically patients with MM represent a minor part of the included patients in exercise studies in haematological cancer populations, and therefore it is unknown if they differ and should be approached separately or with a special focus. The two non-haematological cancer diagnoses (prostate cancer and breast cancer) are the most common gender specific diagnoses and both cancer types share an issue of bone health, bone destructions and bone pain [42]. In the following our study population is called the EMMY population (Exercise in Multiple MYeloma).

### Statistical analyses

Characteristics of the cohort are reported as counts and proportions and stratified by gender. The physical outcome measures 6MWT, SST, grip strength and knee extension strength are reported as mean and standard deviation (SD) and stratified by gender and age groups. Data are compared by z-test (after standardisation to mean = 0 and SD = 1) to reference values from normative populations and furthermore, to published data from patients with malignant lymphoma, prostate cancer and breast cancer, respectively. Moreover, we present outcome measures as box plots stratified by bone involvement and fractures and compare the standardised measurements by Wilcoxon rank sum test. Sample size calculation showed that by inclusion of 100 patients, differences of 0.33 SD with 90% power in the age- and gender-standardised outcome measures, compared to the reference populations, could be detected.

## Results

In the randomised controlled trial, 158 patients were screened for eligibility. Out of the 158 patients, 33 were excluded because they did not meet the inclusion criteria, and 24 declined to participate. One patient accepted, but withdrew and did not give consent to use data. Thereby, the study cohort consisted of 100 participants. Demographic and medical characteristics are presented in Table 1.

**Table 1:** Patient demographics in the total study population and according to gender

Patient characteristics	Total *N* = 100	Male *n* = 58	Female *n* = 42
Age, years			
Mean (SD)	67.7 (10.3)	68.1 (10.7)	67.1 (9.8)
Median (range)	69 (38–90)	70 (38–89)	67.5 (49–90)
Age groups, years (*n* (%))			
≤ 39	1 (1)	1 (2)	0 (0)
40–49	3 (3)	1 (2)	2 (5)
50–59	20 (20)	11 (19)	9 (21)
60–69	28 (28)	15 (26)	13 (31)
70–79	35 (35)	22 (38)	13 (31)
80–89	12 (12)	8 (14)	4 (10)
≥ 90	1 (1)	0 (0)	1 (2)
ECOG performance status^a^ (*n* (%))			
0–1	85 (85)	46 (79)	39 (93)
≥ 2	15 (15)	12 (21)	3 (7)
R-ISS (*n* (%))^b^			
1	21 (21)	9 (16)	12 (29)
2	49 (49)	31 (53)	18 (43)
3	30 (30)	18 (31)	12 (29)
Co-morbidity			
Ischaemic heart disease	6 (6)	6 (10)	0
Incompensated heart disease	6 (6)	6 (10)	0
COL/chronic lung disease	3 (3)	3 (5)	0
Asthma	0	0	0
Rheumatoid arthritis	1 (1)	0	1 (2)
Osteoarthritis	7 (7)	6 (10)	1 (2)
Apoplexia/neurological disease	6 (6)	6 (10)	0
Other	13 (13)	7 (12)	6 (14)
Bone disease	73 (73)	44 (76)	29 (69)
Bone disease with restriction for tests or exercise^c^	41 (41)^e^	23 (40)	18 (43)
Fracture (*n* (%))	33 (33)	19 (33)	14 (33)
Non-vertebral fracture (*n* (%))	9 (9)	3 (5)	6 (14)
Vertebral fracture (*n* (%))	24 (24)	16 (28)	8 (19)
Pain from non-vertebral fracture (*n* = 9)	5 (55)	0	5 (83)
Mild	2 (22)	0	2 (33)
Moderate	1 (11)	0	1 (17)
Functional	2 (22)	0	2 (33)
Pain form vertebral fracture (*n* = 24)	19 (79)	13 (81)	6 (75)
Mild	4 (17)	2 (13)	2 (25)
Moderate	8 (33)	7 (44)	1 (13)
Functional	7 (29)	4 (25)	3 (38)
Pain relieving drugs (*n* (%))			
None	45 (45)	28 (48)	17 (40)
Non-opid/mildly pain relieving drugs^d^	31 (31)	14 (24)	17 (40)
Moderately pain relieving drugs^e^	11 (11)	6 (10)	5 (12)
Strong pain relieving drugs^f^	13 (13)	10 (17)	3 (7)
Walking aid (*n* (%))			
Yes	17 (17)	9 (16)	8 (19)
No	81 (81)	47 (81)	34 (81)
Missing	2 (2)	2 (3)	0 (0)
Working (*n* (%))			
Yes	20 (20)	16 (28)	4 (10)
No	78 (78)	40 (69)	38 (90)
Missing	2 (2)	2 (3)	0 (0)
Working status (*n* (%))			
Working	20 (20)	16 (28)	4 (10)
Retired	56 (56)	29 (50)	27 (64)
Early retirement	3 (3)	1 (2)	2 (5)
Off work sick, full time	14 (14)	8 (14)	6 (14)
Un-employed	1 (1)	0 (0)	1 (2)
On social security	1 (1)	0 (0)	1 (2)
Other reason	2 (2)	1 (2)	1 (2)
Missing	3 (3)	3 (5)	0 (0)

^a^*ECOG* Eastern Cooperative Oncology Group. ^b^*R-ISS* Revised International Staging System. ^c^three participants had restrictions for the test part. ^d^non-opioid drugs. ^e^opioid drugs but less or maximum equivalent to 20 mg morphine per day. ^ef^opioid drugs equivalent to more than 20 mg morphine per day

Mean age (SD) was 67.7 (10.3) years, median (range) was 69 (38–90) years. The age group with the highest representation was 70–79 years (35%), followed by the age group 60–69 years (28%). The major part of the patients (85%) had an ECOG performance status of 0–1. According to the Revised International Staging System (R-ISS), the patients separated into R-ISS1 in 21%, R-ISS2 in 49%, and R-ISS3 in 30%. Comorbidities were rare and mild. Seventeen % were using walking aids. Over half were retired (56%), and 14% were on sick leave. Bone disease was present in 73% of the participants, and in three participants this caused restriction in testing (SST and unilateral knee extension strength) due to femoral bone involvement. Thirty-three per cent had fractures (*n* = 33). Hereof most common were vertebral fractures (73%) resulting in mild pain (17%), moderate pain (33%), and functional pain (29%). Nine per cent had non-vertebral fractures with associated pain that followed the same patterns as the vertebral fractures. In total, 55% used pain relieving medications (31% non-opioid drugs (mild), 11% opioid drugs but less or maximum equivalent to 20 mg morphine per day (moderate), and 13% opioid drugs equivalent to more than 20 mg morphine per day (strong)).

Patients who did not meet the inclusion criteria (*n* = 33), or fulfilled the inclusion criteria but did not wish to participate (*n* = 24) had a similar mean age as the included patients (68.4 years (SD 9.4) and 70.1 years (SD 7.8), respectively), and gender was similar as well (58 and 54% were males, respectively). Around two thirds (67%) of the non-eligible patients and one third (38%) of the patients who did not wish to participate, were screened during hospitalisation. The major part, 94% of the non-eligible patients and 79% of the patients who did not wish to participate, had bone disease, which is slightly more than patients in the study cohort.

**Fig. 1:**
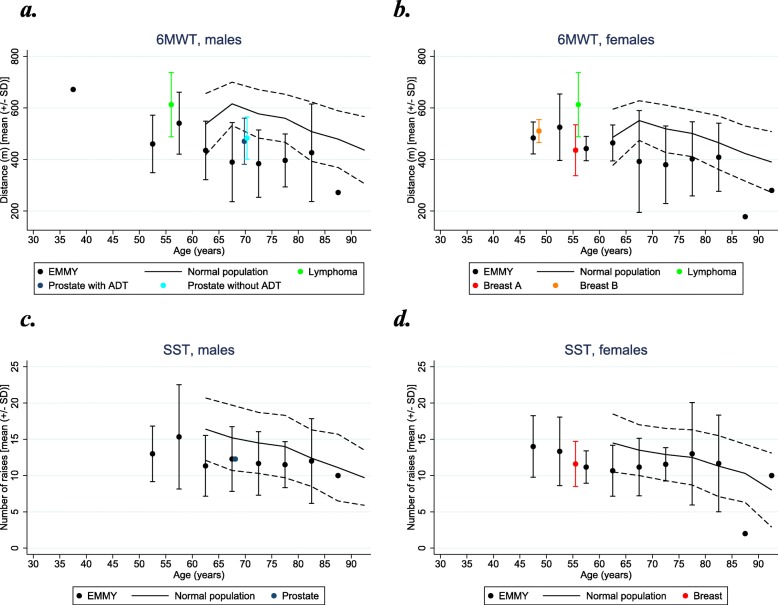
**a**-**d** Age group and gender specific Six-Minute-Walk-Test and Sit-to-Stand-Test in EMMY population, normal population, and cancer populations. **a** Normal [34]. Lymphoma [37], Prostate (+/− ADT) [38]. **b** Normal [34]. Lymphoma [37], Breast A [40]. Breast B [41]. **c** Normal population [34]. Prostate [39]. **d** Normal population [34]. Breast [40]. EMMY data are illustrated by means and SD-bars (within the 5 year intervals) and reference values from the normal populations are illustrated by curves (full line indicates mean and dotted lines are +/− SD)

**Fig. 2:**
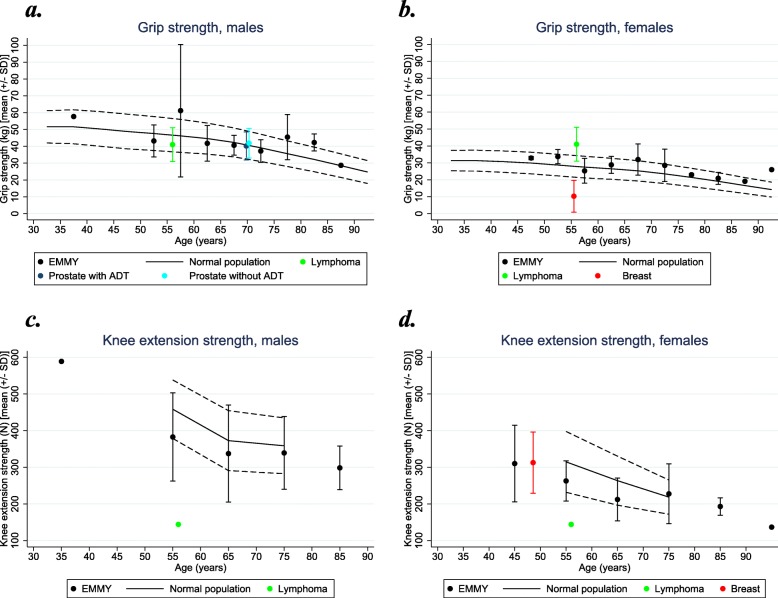
**a**-**d** Age group and gender specific grip and knee extension strength in EMMY population, normal population, and cancer populations. **a** Normal [35]. Lymphoma [37], Prostate (+/− ADT) [38]. **b** Normal [35]. Lymphoma [37]. Breast [40]. **c** Normal [36]. Lymphoma [37]. **d** Normal [36]. Lymphoma [37]. Breast [41]. EMMY data are illustrated by means and SD-bars (within the 5 years intervals for grip strength and 10 year intervals for knee extension strength) and reference values from the normal population are illustrated by curves (full line indicates mean and dotted lines indicate +/− SD)

**Fig. 3:**
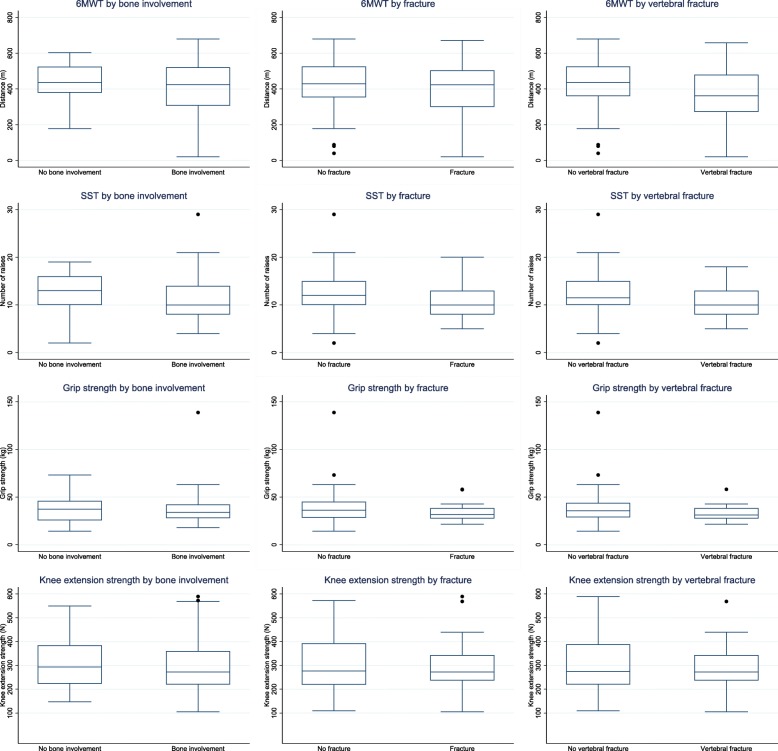
Box plots for Six-Minute-Walk-Test, Sit-to-Stand-Test, and the strength measures according to bone status

The physical function measurement data are presented in Figs. 1a-d and 2a-d, and the specific estimates (mean (SD)) for the four outcome measures are presented in Table 2. Box plots for the four physical measures according to bone disease, fracture and vertebral fracture are presented in Fig. 3.

**Table 2:** Estimates (mean (SD)) for Six-Minute-Walk-Test, Sit-to-Stand-Test, grip and knee extension strength

Gender	Age group	6MWT (distance in meters)		SST (number of raises)		Grip strength (kilograms)		Knee extension strength (Newton)	
		N	Mean (SD)	N	Mean (SD)	N	Mean (SD)	N	Mean (SD)
Males (*n* = 58)	35–39	1	671.75 (.)	0		1	57.70 (.)	1	588.90 (.)
	40–44	0		0		0		0	
	45–49	0		0		0			
	50–54	5	460.24 (111.79)	4	13.00 (3.83)	5	43.20 (9.51)	11	382.71 (120.27)
	55–59	6	540.68 (120.18)	6	15.33 (7.17)	6	61.18 (39.35)		
	60–64	6	435.32 (113.46)	6	11.33 (4.18)	6	41.77 (10.61)	13	337.37 (132.51)
	65–69	9	389.74 (153.62)	7	12.29 (4.46)	9	40.62 (5.92)		
	70–74	12	383.96 (130.49)	12	11.67 (4.38)	13	37.18 (6.73)	21	339.16 (99.37)
	75–79	9	396.43 (102.51)	8	11.50 (3.16)	9	45.46 (13.43)		
	80–84	6	426.44 (189.02)	6	12.00 (5.83)	6	42.28 (5.01)	7	298.39 (59.32)
	85–89	1	272.00 (.)	1	10.00 (.)	1	28.70 (.)		
	90+	0		0		0		0	
Females (*n* = 42)	35–39	0		0		0		0	
	40–44	0		0		0		2	310.05 (104.58)
	45–49	2	483.68 (61.77)	2	14.00 (4.24)	2	32.85 (1.06)		
	50–54	3	525.17 (128.83)	3	13.33 (4.73)	3	33.70 (4.19)	9	262.76 (54.87)
	55–59	6	442.35 (47.28)	6	11.17 (2.23)	6	25.28 (7.33)		
	60–64	4	464.40 (69.78)	3	10.67 (3.51)	4	28.83 (5.02)	10	212.13 (58.41)
	65–69	8	392.34 (197.78)	6	11.17 (3.97)	9	31.98 (9.24)		
	70–74	11	379.78 (150.43)	9	11.56 (2.30)	11	28.50 (9.57)	12	227.53 (81.65)
	75–79	2	402.18 (143.80)	2	13.00 (7.07)	2	23.00 (0.71)		
	80–84	3	408.77 (132.39)	3	11.67 (6.66)	3	20.87 (3.57)	4	192.98 (23.79)
	85–89	1	178.02 (.)	1	2.00 (.)	1	19.10 (.)		
	90+	1	280.00 (.)	1	10.00 (.)	1	26.00 (.)	1	136.60 (.)
Missing (total)		4^a^		14^b^		2^c^		9^d^	

Note: Knee extension strength is reported in 10 year age groups. SD cannot be estimated, if only one observation

^a^Missing data of 6MWT were caused by pain (*n* = 1), sudden impairment (*n* = 2), unknown (*n* = 1)

^b^Missing data of SST were caused by restriction (*n* = 3), pain (*n* = 2), sudden impairment (*n* = 2), personal failure/misunderstanding (*n* = 4), unknown (*n* = 3)

^c^Missing data of grip strength were caused by sudden impairment (*n* = 2)

^d^Missing data of knee extension strength were caused by pain (*n* = 1), restriction (*n* = 1), sudden impairment (*n* = 2), apparatus failure (*n* = 1), personal failure/misunderstanding (*n* = 2), unknown (*n* = 4)

### Six-minute-walk-test (6MWT)

All mean scores, regardless of gender, were lower than for the normal population [34] and furthermore, all mean scores were below the lower SD-reference line for the normal population (Fig. 1a and b). The difference between EMMY and the reference population was statistically significant (*p* < 0.0001, z-score − 1.25). The 6MWT measurement was neither modified by the presence of vertebral fracture (*p* = 0.061), bone disease (*p* = 0.657) nor fracture (*p* = 0.758) (Fig. 3). Compared to lymphoma cancer (mixed genders) aged 55–59 years [37], the EMMY population had a shorter walking distance with a mean difference of 73 m and 171 m for males and females, respectively (Fig. 1a and b). Males with prostate cancer aged 70–74 years [38] achieved a longer walking distance than the EMMY population (Fig. 1a). Females with breast cancer aged 55–60 years [40] had a shorter walking distance than females from the EMMY population (Fig. 1b, Breast B), but younger females with breast cancer (approximately 47 years) [41] had almost the same walking distance as females from the EMMY population (Fig. 1b, Breast A).

### Sit-to-stand-test (SST)

Compared to the normal population [34], males between 60 and 80 years (Fig. 1c) and females between 60 and 75 years (Fig. 1d) had a lower number of mean raises. The total EMMY population (males and females) had statistically significantly lower mean raises than the reference group (*p* < 0.0001, z-score − 0.55), and number of mean raises was modified by the presence of bone involvement (*p* = 0.033) or fracture (*p* = 0.044), but not by vertebral fracture (*p* = 0.058) (Fig. 3). Comparing SST scores for males from the EMMY population to males with prostate cancer within the age group 65–70 years [39] or to females with breast cancer [40] the number of raises was almost identical.

### Grip strength

Grip strength (mean (SD)) in the total group was statistically significantly higher than in the normal population [35] (Fig. 2a and b) (*p* < 0.00001, z-score 0.49) and modified by the presence of fracture (*p* = 0.025) or vertebral fracture (*p* = 0.005), but not bone involvement (*p* = 0.261) (Fig. 3). Compared to the population with lymphoma (mixed group of gender), the females from the EMMY population scored lower than the population with lymphoma cancer, while males had almost the same grip strength [37]. Though, this must be with reservations of comparing a mixed group of gender with females and males, respectively. For males with prostate cancer [38] there was no difference in mean grip strength compared to the EMMY population. Females with breast cancer [40] had a lower grip strength than the EMMY population.

### Knee extension strength

Within the different age groups, the EMMY population (both genders) generally had lower strength compared to the normal population [36] (Fig. 2c and d). For the total group this difference was statistically significant (*p* = 0.0005, z-score − 0.39) and not modified by the presence of bone involvement (*p* = 0.246), fracture (*p* = 0.792) or vertebral fracture (*p* = 0.543) (Fig. 3). The lymphoma population [37] had much lower strength than the EMMY population. Females with breast cancer [41] and the patients from the EMMY population had almost the same strength in the age span 40–50 years.

## Discussion

The aim of this study was to describe age and gender specific physical function among patients newly diagnosed with multiple myeloma and to compare physical function to healthy populations and other cancer populations.

We found that the EMMY population had poorer physical function than the normal population, though unexpectedly, grip strength was found to be better in patients with MM. The presence of bone involvement and fractures modified SST and grip strength (fractures only) and the presence of vertebral fracture marginally modified the 6MWT. In the three cancer comparison groups, we found the patients with lymphoma to have better aerobic capacity, but lower strength in the lower extremities, whereas we did not observe differences compared to the prostate cancer and breast cancer groups, except grip strength, which was better in patients with MM.

Generally, the EMMY population did not follow a clear age-decline pattern. A possible explanation could be that the younger patients (from around 60 years up to 70 years) with MM are more vulnerable to the disease, resulting in affected physical function, than those under the age of 60 and over 70 years, regardless of gender. However, we need to take the number of patients in the EMMY population in each age span into consideration, which means that the uncertainty becomes wider in the younger and older ages. Most patients (63%) were within the ages of 60–79 years. Another explanation could be the confounding factors (bone involvement, fracture or vertebral fracture), which are not related to age. There is no obvious explanation for the better performance in grip strength in the EMMY population compared to the normal population. Possible explanations could be changes in general movement or use of one’s body caused by pain.

Knee extension strength in patients with lymphoma [37] was below the knee extension strength in patients with MM, and accordingly, the grip strength in patients with breast cancer [40] was below the grip strength in patients with MM. According to the authors, the poor knee extension strength might be explained by the disease itself, weight loss as part of B-symptoms, including enhanced protein catabolism, and upregulated tumor necrosis factor stimulating muscle wasting and causing contractile dysfunction [37]. B-symptoms are not common in MM (3). However, it should be added that another study of a mixed group of patients with lymphoma and MM [43] (mean age of 55 years, range 19–67) did not find poorer muscle strength in lower extremities measured by SST [43] compared to the EMMY population.

The poorer grip strength among patients with breast cancer is an expected finding because of disease location and treatment side effects. Further, a study showed that reduced grip strength was not restricted to the affected side [44]. A hypothesis could be that patients with breast cancer generally protect their upper extremities and thus, are losing grip strength. This is underpinned by the comparable results of knee extension strength and SST, respectively between the EMMY population and the breast cancer population. Thus, there does not seem to be a general muscle strength problem among patients with breast cancer.

Patients with lymphoma performed better in the 6MWT compared to patients with MM. In the study by Persoon et al. [43] investigating health-related physical fitness after HDT-SCT, they included patients with MM and patients with lymphoma. Unfortunately, they did not present physical outcome results for the two diagnoses separately, which could either have supported or rejected our interpretation of strength as a challenge for patients with lymphoma and aerobic capacity as a challenge for patients with MM.

### Validity

The Danish test procedure for 6MWT (used in our study) [45] is in accordance with the American Thoracic Society test procedure [24], but Rikli et al. [34] deviated from that procedure regarding instruction to the patient. In the ATS test procedure patients are encouraged to walk as far as possible and are told that they will experience exertion [24], while Rikli et al. [34] told them to walk the best they could, but to avoid pushing themselves to overexertion or beyond what they thought would be safe for them.

Potentially, this could have the consequence that the reference values could be higher, if Rikli et al. [34] had followed the ATS procedure. Thus, the 6MWT difference between the patients with MM compared to reference values may be underestimated. Overall, the test position in the knee extension strength measure does not differ from the one used in the EMMY population. There is a difference regarding grip strength (using sitting or standing position) in the review [35], but the authors conclude that the different positions do not affect grip strength. We assume, that SST is very standardised, and thus does not differ between studies.

### Methods considerations, strengths and limitations

In the field of MM and physical function, the size of our cohort is quite large, and essential characteristics such as age, gender and bone disease are in accordance with the expected in the general MM population [3]. The R-ISS scores were according to the expected [46], although a bit higher proportion (30%) of our participants had R-ISS 3, which probably reflects that our cohort is population based and thereby included more patients with high Beta-2-microglobulin due to renal insufficiency than are observed in randomized clinical trials, because these patients do not fulfil inclusion/exclusion criteria [46]. Comorbidities were rare and mild, probably reflecting that included participants should be able to perform exercise training without being hampered by comorbid condition. Exclusion of patients with comorbidities may have been the physician’s decision as well as the patient’s choice of non-participation. We are not able to provide exact data on that. However, as a consequence, our data reflect the impact of MM and not comorbidities. Though, we have missing data, only a minor part was due to bone disease in the femoral bone (*n* = 3 for the SST). Thus, we believe our study and findings are representative for patients with MM in everyday clinical practice and thereby heighten the external validity of our study.

The associations between physical function and bone disease or fracture, indicate that these subgroups need special attention in a physical function perspective.

It is a strength that we have age specific data from normal samples, but regarding age-specific comparisons, when divided into age groups we are hampered by a rather small number of participants, especially in the lower and upper age groups.

There are some shortcomings in the comparisons, since we were unable to cover the total age span of the EMMY population in the comparisons with the normal population as well as comparisons with other cancer disease populations. We do not have data from citizens under the age of 60 years for 6MWT and SST, and under the age of 55 years for the knee extension strength. However, we assume that the association between age and physical performance will follow the same pattern for the younger age groups (< 60 years) [47], at least, according to the literature, for the walking distance [48, 49] and grip strength [47]. Furthermore, we did not have data on all the needed physical outcomes in the cancer disease populations. Finally, we need to address that the EMMY data are at time of diagnosis, which is different from the time points in the other cancer population studies, except for the lymphoma population. The differences in time points, and settings as well could influence the external validity.

### Implications for practice and future perspectives

Generally, our results indicate, that patients with MM have lower physical function at time of diagnosis and that this particularly is the case for patients with bone involvement. After start of anti-myeloma treatment, physical function may worsen, but we lack strong data on this. Bone studies in MM have shown that early bone fractures are common within the first weeks and observed in about 15% within 3 months [50]. This is assumed to cause deterioration of physical function. Patients undergoing HDT-SCT can experience loss in function during treatment. Potentially, such loss can be prevented or minimised by exercise [51] as shown in exercise studies among other cancer populations [19] and among elderly [20], although little is known about exercise interventions at time of diagnosis [52, 53] . Other treatments than HDT-SCT, typically offered to patients over the age of 65–70 years, are less intensive, but still may affect the physical function as well. Since the patients are older and may be frail [54] early detection of physical decline and subsequent early prevention by providing exercise interventions is of importance.

Our study accommodates the gap of knowledge of physical function in newly diagnosed patients with MM. Although our cohort is relatively large, further research is needed if we want to establish evidence of the physical function limitations. This could have implications for clinical practice, either by identifying patients at risk at group or individual level, and then establish an exercise regimen aiming at preventing physical decline and thereby importantly maintaining independence and quality of life.

## Conclusions

In this Danish cohort of newly diagnosed patients with MM, the patients have reduced physical function compared to the normal population, except for grip strength. In particular, bone disease and fractures influence the physical function.

## Data Availability

The datasets used and/or analysed during the current study are available from the corresponding author on reasonable request.

## Abbreviations

6MWT: Six-minute-Walk-Test
ECOG: Eastern Cooperative Oncology Group
EMMY: Exercise in Multiple MYeloma
HDT- SCT: High Dose Therapy with Stem Cell Transplantation
MM: Multiple Myeloma
SST: Sit-to-Stand-Test
